# Facial Prosthesis

**Published:** 1886-07

**Authors:** 


FACIAL PROSTHESIS.—By C. Edmund Kells, Jr., D. D. S., of New Orleans. This is a small pamphlet containing the reprint from *New Orleans Medical and Surgical Journal*, of an article describing an operation by which an artificial nose was applied in a manner altogether satisfactory to the patient, and exhibiting great skill on the part of the operator.

Illustrations show how a great deformity was happily corrected.

